# Source Robust Non-Parametric Reconstruction of Epidemic-like Event-Based Network Diffusion Processes Under Online Data

**DOI:** 10.3390/bdcc9100262

**Published:** 2025-10-16

**Authors:** Jiajia Xie, Chen Lin, Xinyu Guo, Cassie S. Mitchell

**Affiliations:** 1 Laboratory for Pathology Dynamics, Department of Biomedical Engineering, Georgia Institute of Technology and Emory University, Atlanta, GA 30332, USA; 2 Computational Science and Engineering, Georgia Institute of Technology, Atlanta, GA 30332, USA; 3 Machine Learning Center, Georgia Institute of Technology, Atlanta, GA 30332, USA

**Keywords:** network diffusion, epidemic models, patient zero, temporal diffusion, non-parametric methods, reverse engineering, inverse graph diffusion, source localizations

## Abstract

Temporal network diffusion models play a crucial role in healthcare, information technology, and machine learning, enabling the analysis of dynamic event-based processes such as disease spread, information propagation, and behavioral diffusion. This study addresses the challenge of reconstructing temporal network diffusion events in real time under conditions of missing and evolving data. A novel non-parametric reconstruction method by simple weights differentiationis proposed to enhance source detection robustness with provable improved error bounds. The approach introduces adaptive cost adjustments, dynamically reducing high-risk source penalties and enabling bounded detours to mitigate errors introduced by missing edges. Theoretical analysis establishes enhanced upper bounds on false positives caused by detouring, while a stepwise evaluation of dynamic costs minimizes redundant solutions, resulting in robust Steiner tree reconstructions. Empirical validation on three real-world datasets demonstrates a 5% improvement in Matthews correlation coefficient (MCC), a twofold reduction in redundant sources, and a 50% decrease in source variance. These results confirm the effectiveness of the proposed method in accurately reconstructing temporal network diffusion while improving stability and reliability in both offline and online settings.

## 1. Introduction

A temporal network diffusion process (NDP) models the spread of misinformation [1], behaviors [2], diseases [3,4], or other phenomena over time through a network of interconnected entities. These entities, represented as nodes, can include individuals, molecules, organizations, or devices, while edges define their interactions. Unlike static models that analyze a network at a single time point, temporal diffusion models account for dynamic changes over time.

**Figure 1.**
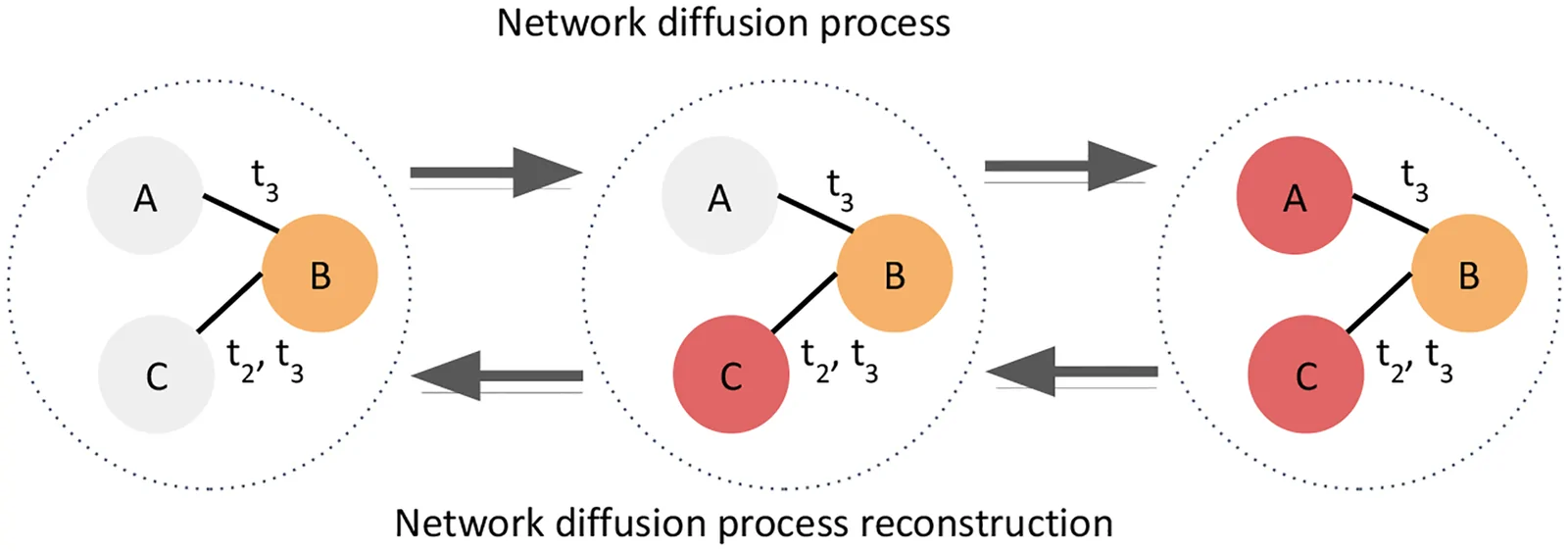
The temporal network diffusion process simulates epidemic-like event behavior within a temporal network (forward). Each activation is an event in red. Its reverse-time process (backward) is the reconstruction of the event-tree or sequence with inferred sources (orange) given the observations from a forward NDP. All gray nodes are either inactivated or hidden activations.

Temporal network diffusion is crucial for predictive modeling in healthcare, social science, machine learning, and information technology. These models capture time-dependent spread patterns, identify optimal intervention points, and enhance resource allocation by targeting key entities at the right time. Figure 1 illustrates the forward diffusion process, which simulates epidemic-like propagation, and its reverse process, also as known as inverse graph diffusion or reconstruction of network diffusion, which reconstructs diffusion with inferred sources.

**Figure 2.**
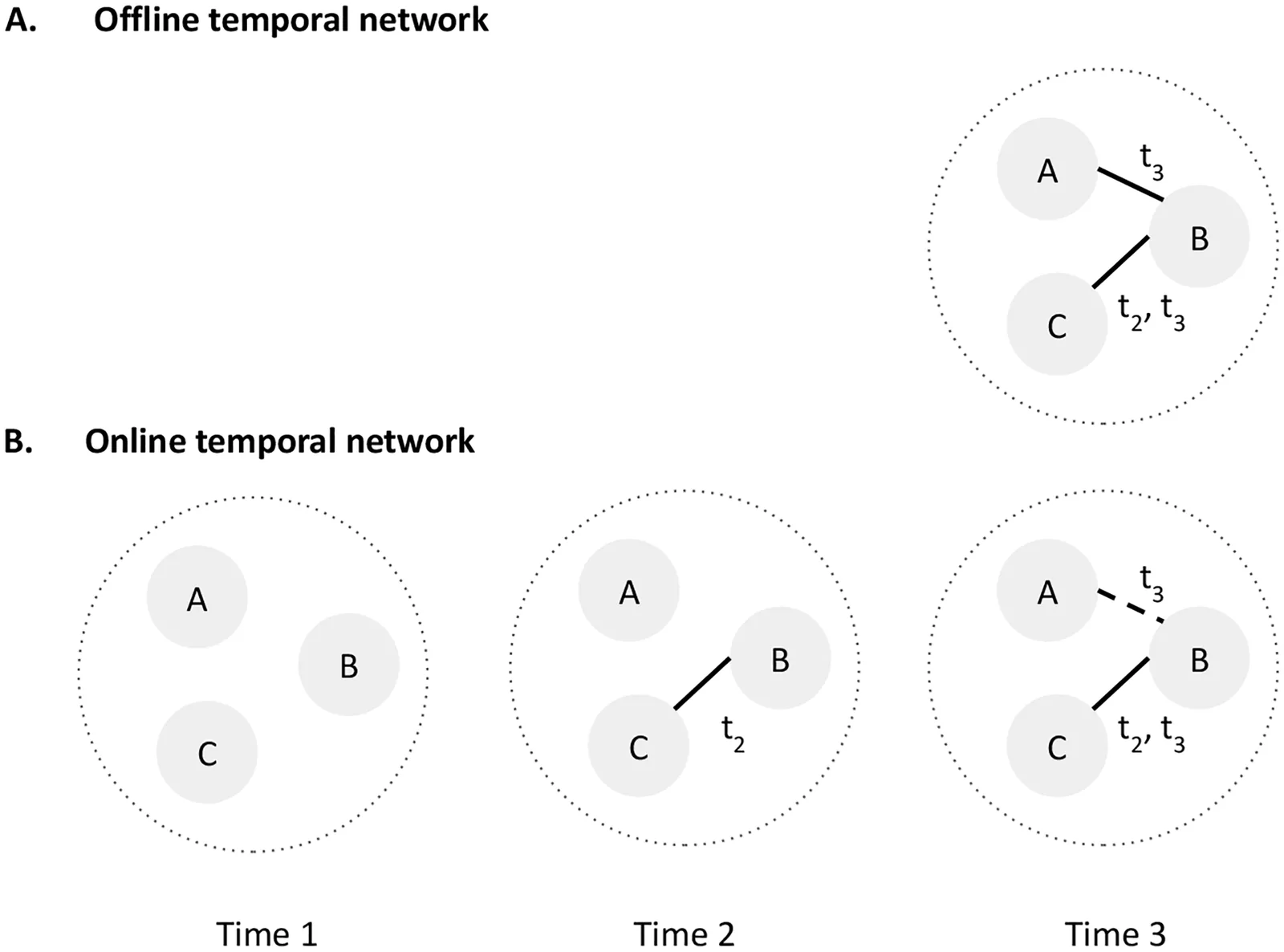
(**A**) Offline temporal temporal network is retrospective data that assumes all information in the past are available and accurate. (**B**) Online temporal constantly updates edges with delay. For instance, at time 3, the edge (dash line) between A and B at time 3 is temporarily missing or uncertain due to delay.

Reconstructing an NDP is essential for reversely engineering the event order or causality through network diffusion and making actionable predictions. This process, which infers the diffusion path from observed data, can be performed offline—where all data is available (Figure 2A), or online, where updates occur in real time. The present study focuses on online reconstruction, using current available edges within a network (Figure 2B). The reconstruction output is a temporal tree preserving chronological activation sequences. Prior research has explored the offline reconstruction of temporal NDP using the temporal Steiner tree formulation [3,5–7], a non-parametric approach that prioritizes minimal sources and shortest paths. Recent work by Mishra et al. reformulated maximum likelihood estimations (MLE) using Steiner connectivity [8], but to our knowledge, online NDP reconstruction remains unexplored.

Constructing real-world temporal networks is expensive and subject to missing data [9–12]. For instance, the contact tracing for a epidemic is extremely time-consuming and challenging given the difficulty to extract information from massive and contaminated information due to constant revision and delay of updates [11]. Online temporal networks constructed for epidemiology, such as using WiFi or 4/5G mobile data for epidemiological contact tracing [11,13], often exhibit missing edges in recent timestamps due to noise, delayed data integration, or other factors. In disease spread models, for instance, some infected individuals may conceal their diagnosis due to stigma, quarantine mandates, or financial concerns [14,15], while test reports may omit asymptomatic or reinfected individuals [16,17]. To address these challenges, online temporal networks require continuous updates to correct missing or erroneous data [18,19].

Similar to contact tracing, many real-world temporal networks observes back-filled edges over time, such as social media’s networks (e.g., Facebook) where friendship can be hidden and can be determined later with additional information or by machine learning models for link predictions [20] and biological/protein network suffering from false negative errors caused by predictive models [21,22]. The online property among these data shares similar sensitivity to missing data and vulnerability to unbounded errors when reconstructing network diffusion for applications ranged from detecting phishing emails [23], identifying cancer driver gene [24], or unveiling pathology dynamics of neuro-degenerative diseases [25].

This work explores the online reconstruction of diffusion processes in dynamically evolving temporal networks, addressing missing data challenges while ensuring robust inference of diffusion sources. We propose a novel and innovative framework that overcomes key existing limitations of the current state-of-the-art online reconstruction methods.

**Figure 3.**
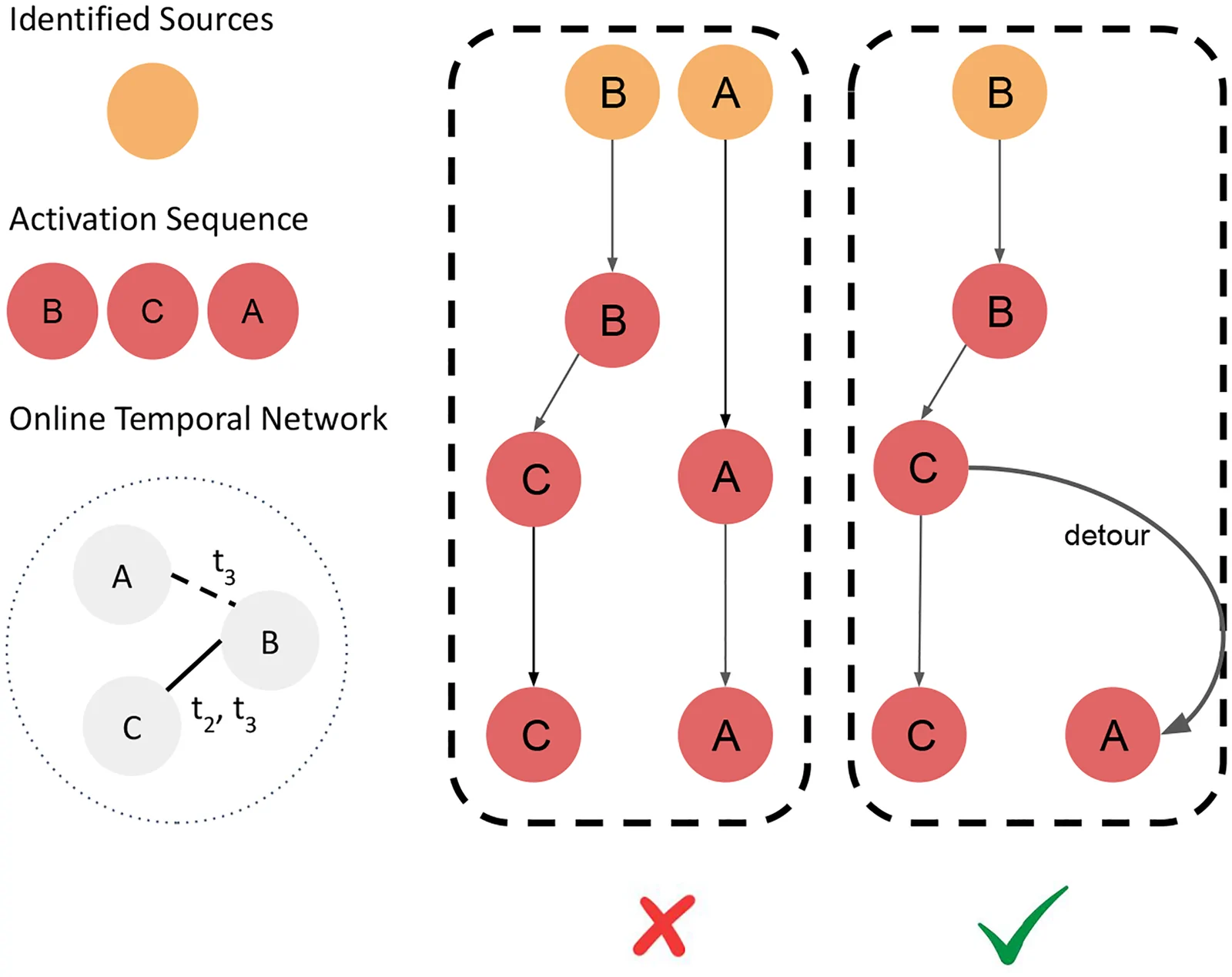
Online reconstruction of NDPs from an online temporal network with missing edges at T=3 (dash edge) given reported activated nodes at all timestamps. The identified sources are colored orange given the activations report (red) in order. The perturbed Steiner trees when all edges at T=3 are missing. Depending on the cost of additional sources, the model may choose A as a redundant source or find a very long detour. The first scenario marked by the red cross sign will generate inconsistent hypotheses, compromising downstream decisions. The second case can violate the parsimonious principle and cause false-positive mistakes. This study proposes that the second scenario is better (the green hook sign) because the false positive errors caused bu detouring can be controlled.

From a practical perspective, offline Steiner-tree-based formulations are inadequate for real-world temporal NDP reconstructions, as illustrated in Figure 3. The model’s sensitivity to temporal network fluctuations causes insufficient control of redundant sources and an elevated false-positive rate. A fundamental challenge arises in deciding whether to detour or introduce a new source, which depends on the number of true sources. This study follows the realistic assumption that a network diffusion starts with a single source, such as every epidemic has an initial patient “zero” [4,8] and aims to minimize the number of inferred sources while controlling false positives introduced by excessive detouring.

Redundant sources—identified sources that are not supported by the data—can compromise downstream tasks such as source localizations. Even small perturbations in network updates can lead to substantial shifts in the inferred diffusion process, undermining previously established hypotheses [26]. These challenges highlight the limitations of existing frameworks that rely on parsimonious principles, which may render models unstable in dynamic settings [3,5,6]. Similarly, methods incorporating Steiner tree sampling within probabilistic models [4,8] remain vulnerable to instability in online settings.

The solution proposed in this study is based on the dynamic cost of source candidates. A high-risk candidate has a lower cost that enables bounded detours from the same previous source; it explains the snapshot with missing edges while maintaining the same source as the past. It is a simple and effective method that takes advantage of the online nature of the problem, where a source identified multiple times in the past is considered high-risk. The main contributions of our work are summarized as follows:

- **Problem Formulation**—A novel online network diffusion reconstruction model is proposed that improves predictions of real-world online temporal networks.
- **Simple Approach** The dynamic costs of high-risk source candidates are evaluated at each step, which creates variation in costs. This enables robust and controllable detours and redundant sources to span the activated nodes over time.
- **Error Bounds**—This study theoretically establishes improved regret bounds on false positives compared to the baseline implementation of the approximation algorithm. This improvement is achieved through weight and cost adjustments, naming it Robust-Ada Sources, ensuring that the adjusted touring reduces errors, see the second minus term shown below. The general improved false-positive error bounds were identified through a regret analysis of the proposed approach.
(1) $$𝒯(T)\leq 𝒪(\text{ln}(T+1)\Psi (T+1))-\underset{\text{Robust-Ada}\;\text{Sources}}{\underbrace{\left(2\alpha -1+(1-\alpha )\delta )\Psi (T\right)}}$$
- **Linearly Model Improvement**—Assuming that positive cases follow logistic growth. Without weight adjustment, the additional errors grow linearly and are always greater than or equal to those observed with adjustment, provided that the regularization parameter δ>2, is shown below.
(2) $$\underset{\text{baseline}}{\underbrace{\left(\lambda -1)𝒪(T\right)}}\geq \underset{\text{Robust-Ada}\;\text{Sources}}{\underbrace{\left(\lambda -2\alpha +1-\delta (1-\alpha ))𝒪(T\right)}}$$
- **Empirical validation**—Empirical experiments demonstrate that the novelly proposed approach exhibits greater robustness and consistency in both offline and online settings. Results indicate an average twofold reduction in redundant sources and a 50% decrease in source variance. Additionally, the approach achieved a 5% increase in Matthews correlation coefficient (MCC) accuracy by reducing false positives in the presence of missing edges across various scales.

## 2. Related Works

The reconstruction of a network diffusion process (NDP) is closely related to the “reverse-engineering” of cascades. For example, Lappas et al. studied identifying k sources from a partially activated network assumed to be in steady state under the independent-cascade (IC) model. Shah and Zaman introduced rumor centrality to identify a single “culprit” under the Susceptible–Infected (SI) model [27]. Prakash and Sundaresan proposed a formulation based on the Minimum Description Length (MDL) principle under the SI model, which can automatically select the number of sources and tolerate false-negative reports within a snapshot [28,29]. However, these approaches are model-specific and do not provide a universal formulation for temporal network data.

To the best of our knowledge, Rozenshtein et al. were the first to formulate the problem non-parametrically on temporal networks using the Minimum Directed Steiner Tree, yielding a model-agnostic approach that accommodates multiple sources and missing infections [5]. Many subsequent works extend this framework: Xiao addressed settings with exact activation times [3]; Jang et al. extended to healthcare-associated infections involving human–object interactions and incorporated risk augmentation [6,7]. The Steiner-tree perspective also connects to maximum-likelihood estimation: Makar et al. tackle latent false positive cases via MLE [30], and Mishra reformulates MLE using Steiner connectivity [8].

Recent advances in learning-based diffusion inference typically employ over-parameterized models to learn the propagation mechanism from data. While effective for retrospective prediction, these approaches often under-address the inherently online nature of temporal networks and provide limited first-principles guarantees. For example, invertible-aware graph diffusion inverses sources via an invertible residual architecture [31]; variational Bayesian formulations target uncertainty quantification over source candidates [32]; and heterogeneous hypergraph attention networks model multi-pathway transmission in mixed populations [33]. However, such methods typically (i) rely on large amounts of simulated or historical training data, (ii) require stationary or slowly drifting dynamics during training–deployment, and (iii) prioritize comparative accuracy over model-agnostic control of error amplification in streaming settings. In contrast, our first-principles approach explicitly analyzes and mitigates sensitivity at its root—by adapting source costs online—yielding provable false-positive bounds for Steiner-tree-based non-parametric methods and consistent behavior without assuming a parametric diffusion law or abundant labeled trajectories.

This study extends Steiner-tree–based non-parametric reconstruction to a source-robust formulation for online temporal networks. Prior work has targeted robustness to partially observed activations [4] incorporated attribute-based risk factors [6], or for source detections with offline incomplete nodes instead edges [34]. To our knowledge, online reconstruction under incomplete networks at the latest timestamp has not been explicitly addressed.

## 3. Materials and Methods

Our methodology relies on differentiating the costs of source candidates while attaining a minimum-directed Steiner tree from the time-expanded network at every timestamp. More precisely, the cost gap between high-risk and low-risk candidates is designed to widen over time. This gap encourages the minimum directed Steiner tree to remain rooted at high-risk sources even as the temporal network evolves online.

### 3.1. Notations

We summarize the symbols used throughout the paper for clarity in Table 1.

### 3.2. Minimum Directed Steiner Tree

**Figure 4.**
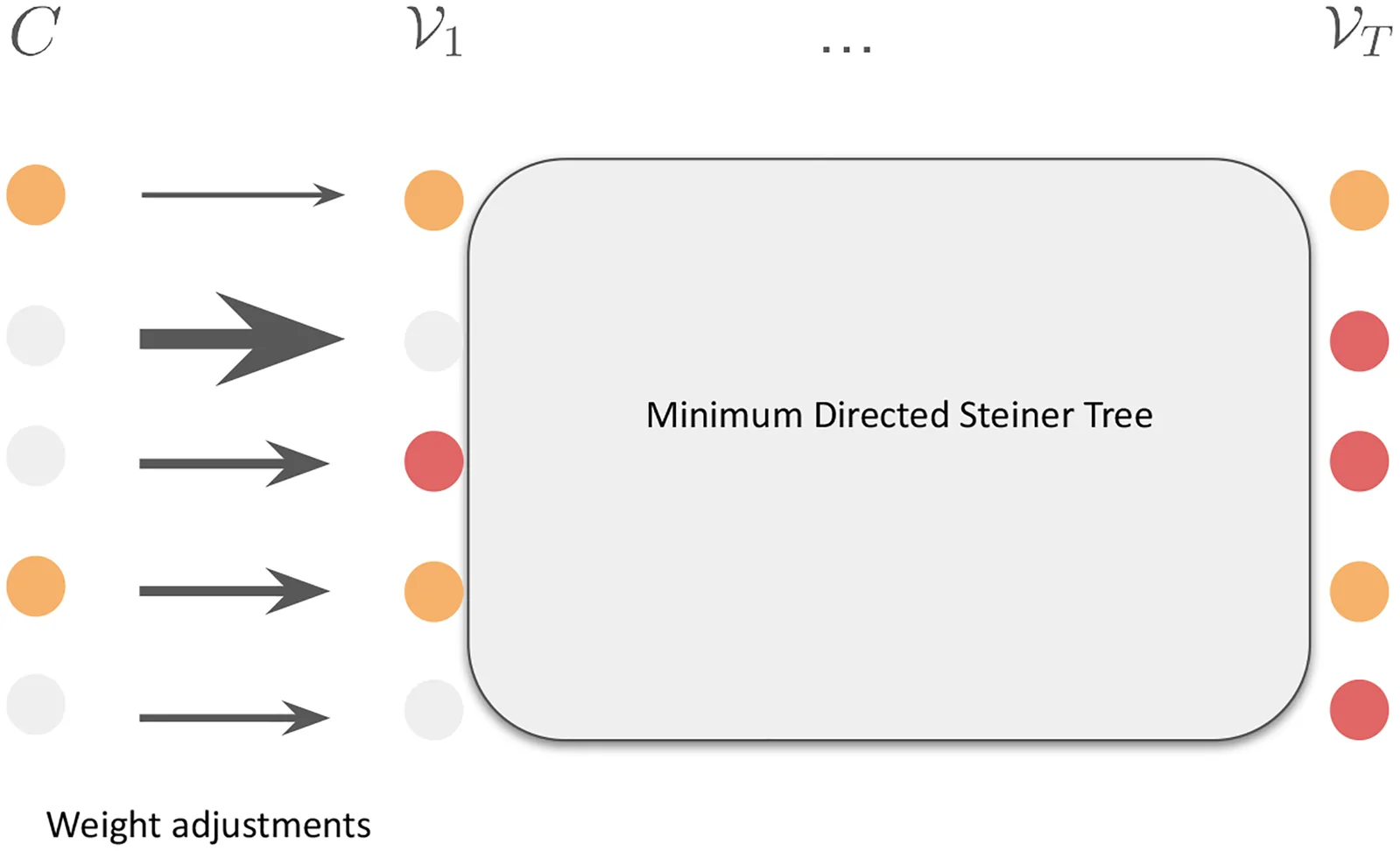
Schematic of the proposed weight adjustment at each timestamp. We differentiate source-candidate costs to promote robust temporal trees to span those activated nodes (red) in chronological order so that the first layer between the source candidates and the first timestamp of the temporal network has differentiated edges’ weight (reflected by the arrow size) with the high-risk source candidates (orange) smaller. The framework is generalizable to non-parametric methods that approximately solve Minimum Directed Steiner formulations on the time-expanded network.

We adopt the standard Minimum Directed Steiner Tree formulation for non-parametric reconstruction of NDPs [5]. This problem is a well-known NP-hard problem [17]. The best approximation ratio is $𝒪\left(\ell (\ell -1)K_{t}^{1/\ell }\right)$ for recursion parameter ℓ≥2 and $K_{t}$ activated nodes. In our experiments we use a level-2 (ℓ=2) linear-programming heuristic; importantly, the proposed weight adjustments are compatible with any Steiner-tree–based approximation (Figure 4).

### 3.3. Our Weight-Adjustment Framework: Robust-Ada-Source

By increasing the cost gap, the model continues to favor high-risk sources even if short detours are required under missing edges. In this study, “high-risk” means candidates previously inferred as sources; extending this to other empirical risk definitions is straightforward [6]. Formally, at time t let $C_{t}^{H}\subseteq C$ denote the high-risk set. For illustration, consider the case where all nodes have incident edges at t=1. We attach a dummy node $z_{c}$ to each $c\in C_{t}^{H}$ and assign a time-varying dummy-to-candidate cost that decays with t:

(3) $$w_{t}^{\left(z_{c},1\right),(c,1)}\leftarrow \text{max}\left(\frac{w_{t-1}^{\left(z_{c},1\right),(c,1)}}{t},\delta \right),\;c\in C_{t}^{H}.$$

This update decays the source-opening cost by a factor of t while enforcing a positive floor δ>0 to avoid vanishing weights. We initialize all dummy-to-candidate costs at t=1 to the constant λ>0, a standard choice that discourages spurious sources [5,6]. With these definitions, we summarize the procedure in Algorithm 1.

**Figure 5.**
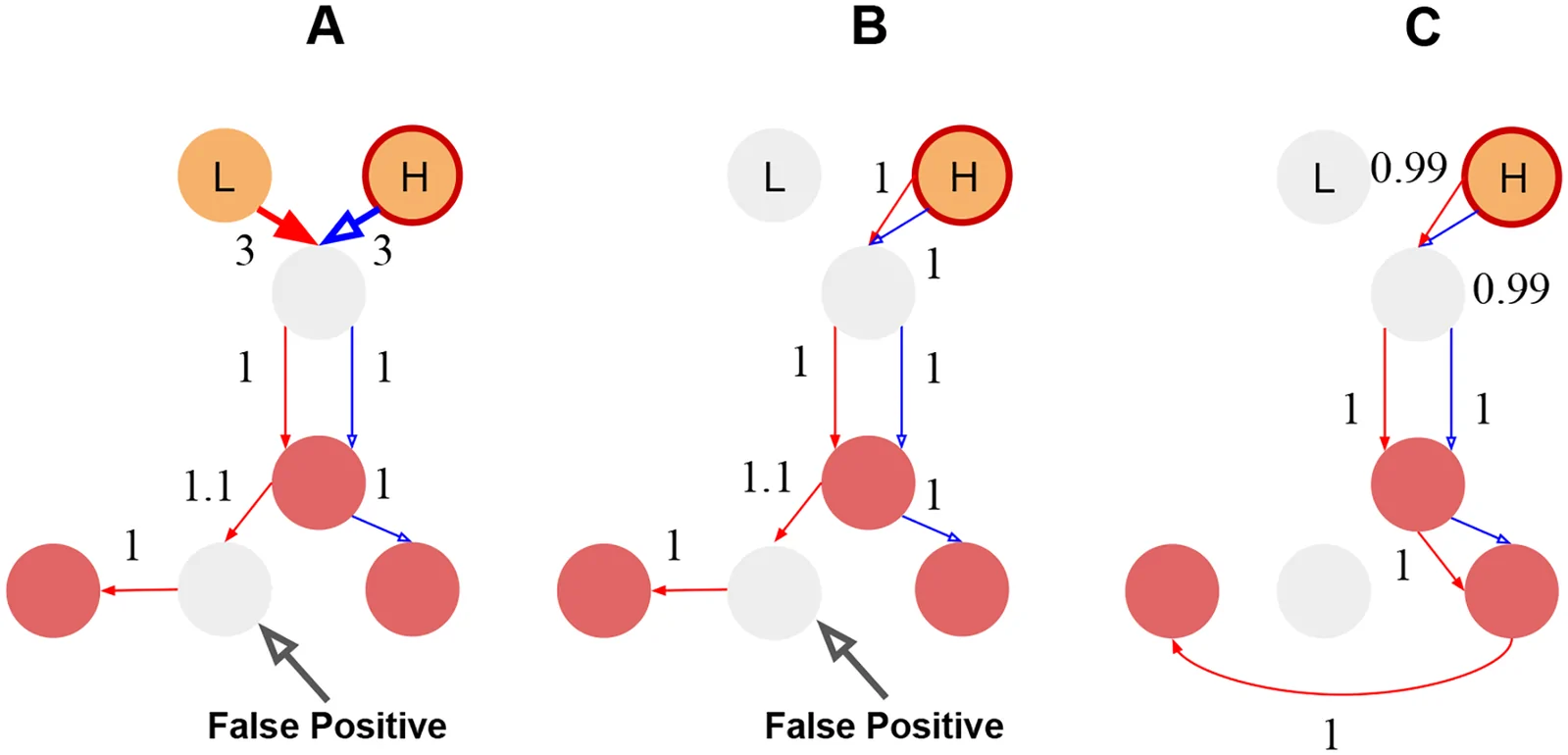
A toy example of level-2 approximation with *Robust-Ada-Source* to demonstrate how to reduce redundant sources (**B**) and false positive (**C**). Selected sources are colored orange. Two source candidates are shown with one lower-risk source candidate labeled as L and the other high-risk candidate as H. The current activated nodes are in red color. The goal is to find shortest paths as a Steiner tree approximation to span the red activated nodes. Shortest paths are arrows colored in either red or blue respectively. (**A**) is the baseline comparison without weight adjustments on the source candidates. The level-2 approximations will combine two shortest paths from different sources as the Steiner tree approximation. (**B**) is the *Robust-Ada-Source* which decays the cost of high-risked (labeled as H) source candidate as 1. The two shortest paths share the same source H to approximate the Steiner tree. The likely redundant source L is thereby not selected by the tree. (**C**) further decreases the high-risk’s candidate’s cost to be 0.99 encouraging longer detour from the same H source which successfully bypass a False Positive error as highlighted in (**A,B**). When the data is online, the 1.1 cost of edge to the false positive node represents the uncertainty of missing this edge which causes the algorithm to bypass given the smaller initial weight of H.

The intuition behind differentiating source candidate costs is the eliminations of identical-distance paths given online data. Observed that even without edge dropout, any level-2 approximation cannot distinguish single and multiple-source Steiner trees when their total distance equals each other. We demonstrated the difference and the effectiveness of Algorithm 1 using a toy example as shown by Figure 5. Figure 5A is the standard case with a zero-cost gap between low-risk and high-risk candidates. The level-2 approximation will therefore look for a tree with two sources to span all red activated nodes. Figure 5B show an immediate effect after decaying the cost of the high-risk candidate to 1. The tree turns to root purely at the high-risk candidate. If we further decay this cost, the tree’s root will remain but encouraging longer detour Figure 5C to bypass the potentially missing edge (we use 1.1 for this case to differentiate from certain edges with cost as constant 1). For this example, we are able to eliminate one false positive error.

**Algorithm 1:** Online Algorithm

**Input:** C **Parameter:** λ,T,δ	
1:	Initialize $R_{0}$ as an empty set.
2:	**for** c∈C **do**
3:	$t_{c}=\text{arg}\;{\text{min}}_{(c,t)\in V}\;t$
4:	Initialize all $w_{1}^{\left(z_{r},t_{r}\right),\left(r,t_{r}\right)}\leftarrow \lambda$
5:	**end for**
6:	**while** t≤T **do**
7:	$C_{t}\leftarrow C$
8:	Given $G_{1}\ldots G_{t}$ and an instant report set $\nabla_{t}$.
9:	Construct the static network ${𝒢}_{t}=\left({𝒱}_{t},{ℰ}_{t}\right)$.
10:	Take the union of reports $R_{t}=R_{t-1}\cup \nabla_{t}$.
11:	**for** $c\in C,r\in 𝒰\left(R_{t}\right)$ **do**
12:	**if** c,r has no path **then**
13:	$C_{t}\cup \{r\}$
14:	Add dummy nodes $\left(z_{r},t_{r}\right)$ to ${𝒱}_{t}$
15:	Add edge $<\left(z_{r},t_{r}\right),\left(c,t_{r}\right)>$ to ${ℰ}_{t}$
16:	Initialize $w_{t}^{\left(z_{r},t_{r}\right),\left(r,t_{r}\right)}\leftarrow \lambda$
17:	**else**
18:	Compute the shortest path ${\overset{‾}{P}}_{t}^{c,r}$ and ${\overset{‾}{D}}_{t}^{c,r}$
19:	**end if**
20:	**end for**
21:	$x^{t}\leftarrow \text{Solving}\;\text{minimum}\;\text{directed}\;\text{Steiner}\;\text{tree}$.
22:	Update $w_{t+1}$ based on Equation (3).
23:	**end while**
24:	**return** $x_{T}$

The above observation further inspires various source candidates to rule out equivalent multiple-source candidates which enhance robustness. The only concern is how many of new false positive errors will be introduced by the over-detouring as seen in Figure 5C. To evaluate this property of our approach, *Robust-Ada-Source*, we analyze the regret for our online model, which is typically used by online optimization literature, between offline and online NDP reconstruction [35]. The regret in our case is the maximum detour to span reports and thus can be interpreted as the number of extra identified activated or positive in terms of classification cases. This success benchmark is defined as:

(4) $$𝒯(T)=\sum_{t=1}^{T}f\left(x|{\overset{‾}{P}}_{t},{\overset{‾}{D}}_{t}\right)-\underset{x^{t}\in 𝒳}{\text{min}}f\left(x_{t}|P_{t},D_{t}\right)$$

Other parameters not related to missing edges are omitted. One theoretical contribution of this work is to show that *Robust-Ada-Source* even with detouring will enjoy less false positive errors.

### 3.4. Our Level-2 Approximation Heuristic

Since the minimum directed Steiner tree formulation is NP-hard, a common practice is to design approximation heuristics reaching the golden benchmark approximation ratio $𝒪\left(\sqrt{K_{t}}\right)$ [3–5]. We present our $𝒪\left(\sqrt{K_{t}}\right)$ approximation heuristic for the minimum directed Steiner tree. The heuristic is based on solving a linear programming problem with a polynomial number of constraints that combine the shortest paths to span all activated nodes in expanded temporal networks. First, let and $P_{t}=\left\{P_{t}^{c,r}|\forall c\in C,\forall r\in 𝒰\left(R_{t}\right)\right\}$ and $D_{t}=\left\{D_{t}^{c,r}|\forall c\in C,\forall r\in 𝒰\left(R_{t}\right)\right\}$ be all of the shortest path sets and the corresponding distance between candidates and reports. Our online algorithm relies on solving this linear programming problem, denoted by $f\left(x|{\overset{‾}{P}}_{t},{\overset{‾}{D}}_{t},w_{t},R_{t},C\right)$, at each step.

(5) $$\underset{x}{\text{min}}\;\sum_{c\in C}\sum_{r\in 𝒰\left(R_{t}\right)}x^{c,r}{\overset{‾}{D}}_{t}^{c,r}\;\text{s.t.}\;\sum_{{\overset{‾}{P}}^{c^{\prime },r^{\prime }}\in {\overset{‾}{P}}_{t}}x^{c^{\prime },r^{\prime }}1\left(r\in {\overset{‾}{P}}^{c^{\prime },r^{\prime }}\right)\geq 1,\forall r\in 𝒰\left(R_{t}\right)\;0\leq x^{c,r}\leq 1,\forall c\in C,\forall r\in 𝒰\left(R_{t}\right)$$

Note that the shortest paths are their distance ${\overset{‾}{P}}_{t},{\overset{‾}{D}}_{t}$ are assumed to be compromised by missing edges in the last timestamp. The above minimization problem defined in Equation (5) approximates the Steiner Tree by concatenating the shortest paths between the candidates and the targets at the minimum distance inspired by [3,5,6]. Although this is a linear programming formulation, the solution is guaranteed to be integer, i.e., x∈{0,1}. We establish the theorems and proofs in Appendix B Corollary A1.

## 4. Theoretical Results

This section establishes rigorous bounds on false-positive errors for *Robust-Ada-Source* and the baseline. We prove that the false-positive regret of *Robust-Ada-Source* is strictly lower than that of the no-adjustment baseline under mild assumptions. Counterintuitively, enabling bounded detours leads to *fewer* errors than opening new sources, consistent with Figure 3.

### 4.1. General Upper Bound on Additional False Positives

Our main result (Theorem 1) provides a general upper bound on the additional false positives incurred online relative to the offline optimum. We quantify this via the regret, defined as the excess number of events incorrectly classified as activated by the online method compared with the offline solution. Because the initialization λ>0 is chosen large to discourage spurious sources, using an identical λ for all candidates makes the baseline sensitive at the last timestamp: it tends either to introduce a redundant source or to take a detour whose cost approaches that of adding a new source. By contrast, our dynamic costs widen the gap between high- and low-risk candidates, thereby reducing the frequency and magnitude of such errors.

**Theorem 1** (General Regret Bound). *Let* $\psi (t)=\left|R_{t}\right|$ *denote the cumulative number of reports at time* t. *Suppose the missing edges induce a false positive set* $S_{t}$ *of sources with size*:

$$\left|S_{t}\right|=\alpha \psi (t),\;\text{for}\;\text{some}\;0<\alpha <1.$$

*Assume further that:*

(6) $$\frac{2\alpha -1}{\alpha -1}\leq \delta .$$

*Then, the cumulative false positive regret* 𝒯(T) *of the proposed Robust-Ada-Source algorithm satisfies:*

(7) $$𝒯(T{)}^{\text{Robust-Ada-Source}}\leq \lambda \;\text{ln}(T+1)\Psi_{1}(T+1)-[2\alpha -1+(1-\alpha )\delta ]\Psi_{0}(T)$$

*For comparison, a baseline method satisfies*:

(8) $$𝒯(T{)}^{\text{baseline}}\leq [\alpha \lambda \;\text{ln}(T+1)+1-\alpha ]\Psi_{1}(T+1)$$

*Moreover, under mild conditions discussed in the proof, we have*:

$$𝒯(T{)}^{\text{Robust-Ada-Source}}\leq 𝒯(T{)}^{\text{baseline}}.$$

The above theorem states that our method can best attain sublinear regret if the integral of the reports also grows sub-linearly. In addition, *Robust-Ada-Source* has lower false positive errors compared to the baseline with minor assumptions. To show the generalizability of Theorem 1, we present a special case about Ψ(T) and regularization δ to determine the superiority of Robust-Ada-Source. The proof can be found in Appendix B.1.

### 4.2. A Case Study on Logistic Growth with Independent Parameter δ

We present a real-world scenario for a linear regret when the report size grows logistically. This scenario is realistic and simple to implement because it further refines the regularizer hyper-parameter as any constant greater than 2 without considering the infeasible reports ratio α due to missing edges. Logistic curves are standard epidemiology models with environmental constraints [16]. The accumulation (integration) of logistic curve is in linear order 𝒪(T). We claim that the logistic curve will yield reduced errors for *Robust-Ada-Source* as long as the regularizer δ>2, which is a tighter requirement of $\delta >\frac{2\alpha -1}{\alpha -1}$. This shows a special case holds under the general Theorem 1 and suggest we can use any δ>2 without assuming α.

**Theorem 2.** *In additions to the same assumptions of Theorem 1, for each* t∈[T], *assuming the instant size of reports grows logistically:*

$$\psi (t)=𝒪\left(\frac{1}{1+\text{exp}(-\tau (t-T/2))}\right)$$

*a typical* S-*shape growth of activations [11,13]*. τ>0 *is the growth rate. The upper bounds on the baseline and Robust-Ada-Source are as follows:*

1. *Baseline*:
(9) $$𝒯(T{)}^{\text{baseline}}=(\lambda -1)𝒪(T)$$
2. *Robust-Ada-Source*:
(10) $$𝒯(T{)}^{\text{Robust-Ada-Source}}=(\lambda -2\alpha +1-\delta (1-\alpha ))𝒪(T)$$
  (9) ≥ (10) *as long as* δ>2.

Theorem 2 implies that the maximum additional false positive cases of the dynamic cost in high-risk candidates under missing edges are always smaller than the baseline of the counterpart if the regularizer δ>2 further tightens the requirement as $\delta \geq \frac{2\alpha -1}{\alpha -1}>\frac{2\alpha -2}{\alpha -1}=2$. While the regret between errors is in a linear order, no algorithm can be better than 𝒪(T) by Theorem 1 because Ψ(T)=𝒪(T). The proof can be found in the Appendix B.2.

## 5. Experimental Results and Discussion

While Theorem 1 ensures bounded false positive cases of our approach, a model is not necessarily better off because of improved classification metrics unless fewer source numbers are found. For instance, we can have as many sources as the size of reports to have perfect precision and recall without controlling the source number. Thus, we further evaluate the performance of our model against the standard baseline non-parametric method on real-world temporal networks with two hypothetical network diffusion models under various conditions on growth and perturbations. It is challenging to attain 100% true real-world NDP considering the definition of false positive [29,30].

### 5.1. Experiments Setup

#### 5.1.1. Temporal Networks

We select three temporal networks to represent different epidemic topologies. (1) copresence-SFHH, a physical-temporally collocation network of an African village for simulating respiratory infectious diseases such as COVID-19 [36], (2) fb-messages, a social temporal network based on Facebook messages for simulating social influence [37], and (3) escort, a sexual network between Brazilian escorts and buyers for modeling sexually transmitted disease such as HIV [16]. The summary statistics of these data are described by Table 2:

#### 5.1.2. Diffusion Models and False Positive

We experiment with two standard network diffusion models: (i) susceptible infected (SI); (ii) independent cascade (IC); We follow the temporal activation assumption that the activated nodes will be reported shortly after the exact infected timestamp [3]. To simulate false positive cases, at each round, we sample 20% of the accumulated reports $R_{t}$ and remove them. We set the transmission probability ρ=0.7. The reports will be continuously released after the first timestamp. We sampled 3, 5, and 10 sources to trigger epidemics at t=0 for the three datasets, copresence-SFHH, fb-messages, and escort. We let C be the set of nodes of $G_{0}$.

#### 5.1.3. Comparable Approaches

We compare two general non-parametric approaches for $𝒪\left(\sqrt{K_{t}}\right)$ approximating Steiner trees.

1. ***Baseline:*** a standard level-2 shortest paths approximation [3,5].
2. ***Robust-Ada-Source:*** the same approximation implemented by Algorithm 1 with the dynamic costs defined in Equation (3).

For simplicity, we set λ=5000 and δ=10 throughout the study for the two approaches. We emphasize that while various approximation algorithms for non-parametric NDP reconstruction, e.g., Greedy and delayed-BFS [3], Steiner Tree sampling [4], Prize-collecting Steiner tree [6] exists, they stem from the main Directed Steiner Tree problem implying each algorithm must have an equivalent online formulation as the minimization problem defined in Equation (5). We leave the exploration of more online formulations and seek a further generalizable optimization framework integrating dynamic costs of high-risk sources to those variations as future work.

#### 5.1.4. Cross-Edges Dropout

We simulate real-world noisy temporal networks with randomly sampled dropout edges from the last $G_{t}$ given $G_{0},G_{1}\ldots G_{t}$ for every t to simulate real-world online temporal network constructions. At every t, we sample 20%, and 80% of the cross-edges and remove them from $G_{t}$. In addition, we add 0% as an edge case for investigating the effectiveness of our method with complete offline data.

#### 5.1.5. Metrics

To evaluate the quality of the solution, temporal Steiner trees, we compare the set of nodes in the Steiner trees with a ground-truth set of activated nodes. We use the Matthews correlation coefficient (MCC) defined as follows:

(11) $$\text{MCC}=\frac{\text{TP}\times \text{TN}-\text{FP}\times \text{FN}}{\left(\text{TP}+\text{FP})(\text{TP}+\text{FN})(\text{TN}+\text{FP})(\text{TN}+\text{FN}\right)}$$

where TP, FP, TN, and FN are true/false positive, and true/false negative, respectively. We also compare the number of sources identified by the models as superfluous sources, indicating a violation of the parsimonious principle. An ideal non-parametric method should control the number of sources while maintaining decent MCC. Last, the variance of sources over time directly informs the degree of inconsistency. Because the current missing edges will be available at the next timestamp, redundant sources can enlarge the variance of source numbers if the results disagree.

### 5.2. Analysis

**Figure A1.**
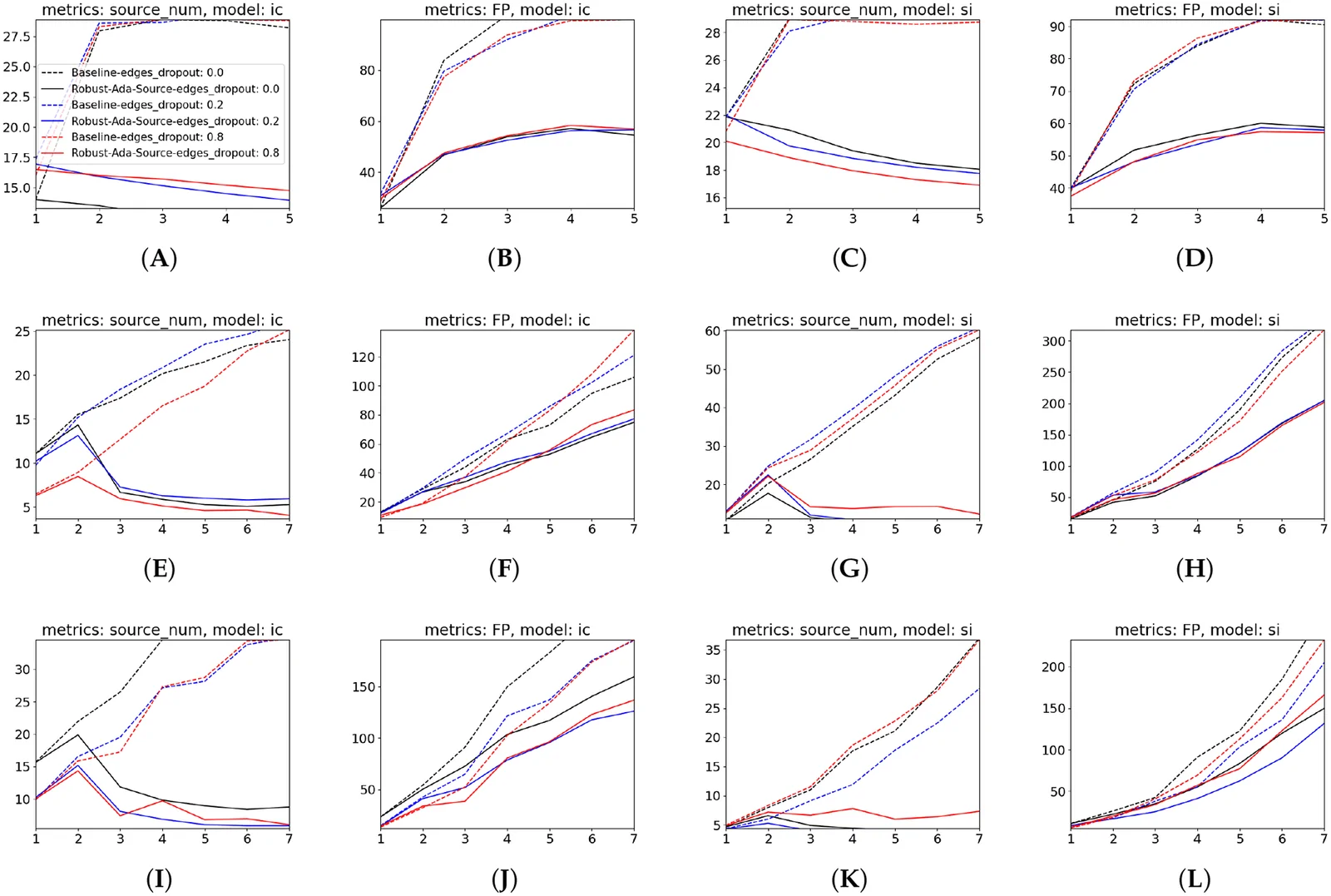
Source counts and false positives (FP) over time for three datasets under two diffusion models with 20% dropout of reports. Rows correspond to datasets: Copresence–SFHH (**A–D**), fb-messages (**E–H**), and escort (**I–L**). Within each dataset, the left pair shows IC (source counts then FP) and the right pair shows SI (source counts then FP). Each line is the mean over 20 runs; the X-axis is the timestamp.

Overall, the results on three real-world temporal networks suggest a raised average MCC by more than 5% with decreased redundant sources by almost two times against the baseline under both online and offline scenarios. Furthermore, the inconsistency of sources has been released as measured by the relatively small variance (50% lower) of sources over time. Except for the main figures present in this secton, the comprehensive results in table can be found in Appendix C Table A1 and Appendix C Figure A1.

#### 5.2.1. *Robust-Ada-Source* Outperforms All of the Baselines by Controlling Source Numbers over Time Under Various Hypothetical Growths of Positives and Perturbations

**Figure 6.**
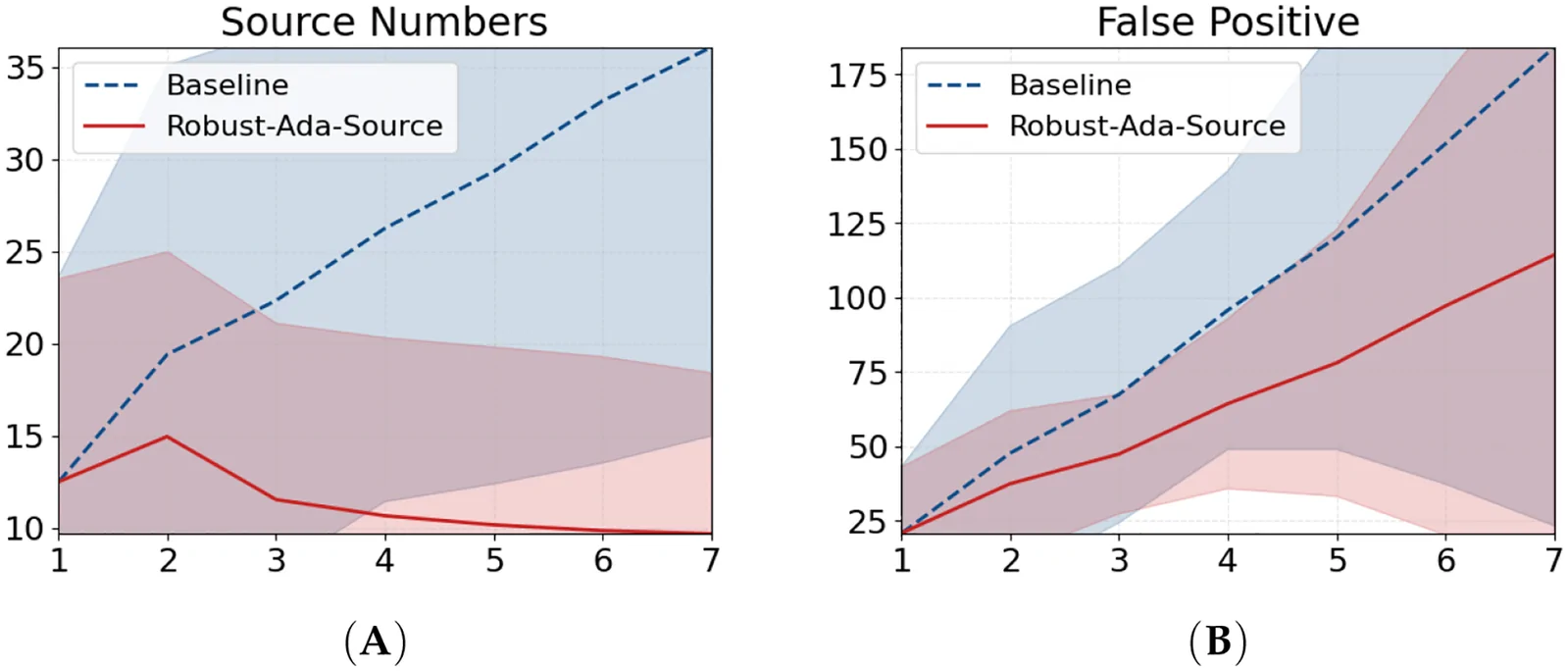
(**A**) Source-count trajectories over the online experiment (lower is better: fewer redundant sources and more consistent trees). Each line shows the mean with 95% confidence intervals over 20 runs, aggregated across the three datasets; the X-axis is the timestamp. *Robust-Ada-Source* (red) maintains a controlled trajectory that stabilizes at 10 sources near the end, whereas the baseline (blue) grows approximately linearly and remains unbounded. (**B**) False positives (FP; lower is better) under the same setting as (**A**). *Robust-Ada-Source* (red) enforces sublinear growth in FP, while the baseline (blue) increases roughly linearly. Notably, the lower bound of the 95% confidence interval for *Robust-Ada-Source* decreases over time, approaching 25 FP by the end. Overall, *Robust-Ada-Source* effectively controls both source counts and false positives throughout the online experiments, yielding more stable and accurate reconstructions. The breakdown results across all datasets and models can be found in Appendix C Figure A1.

**Figure 7.**
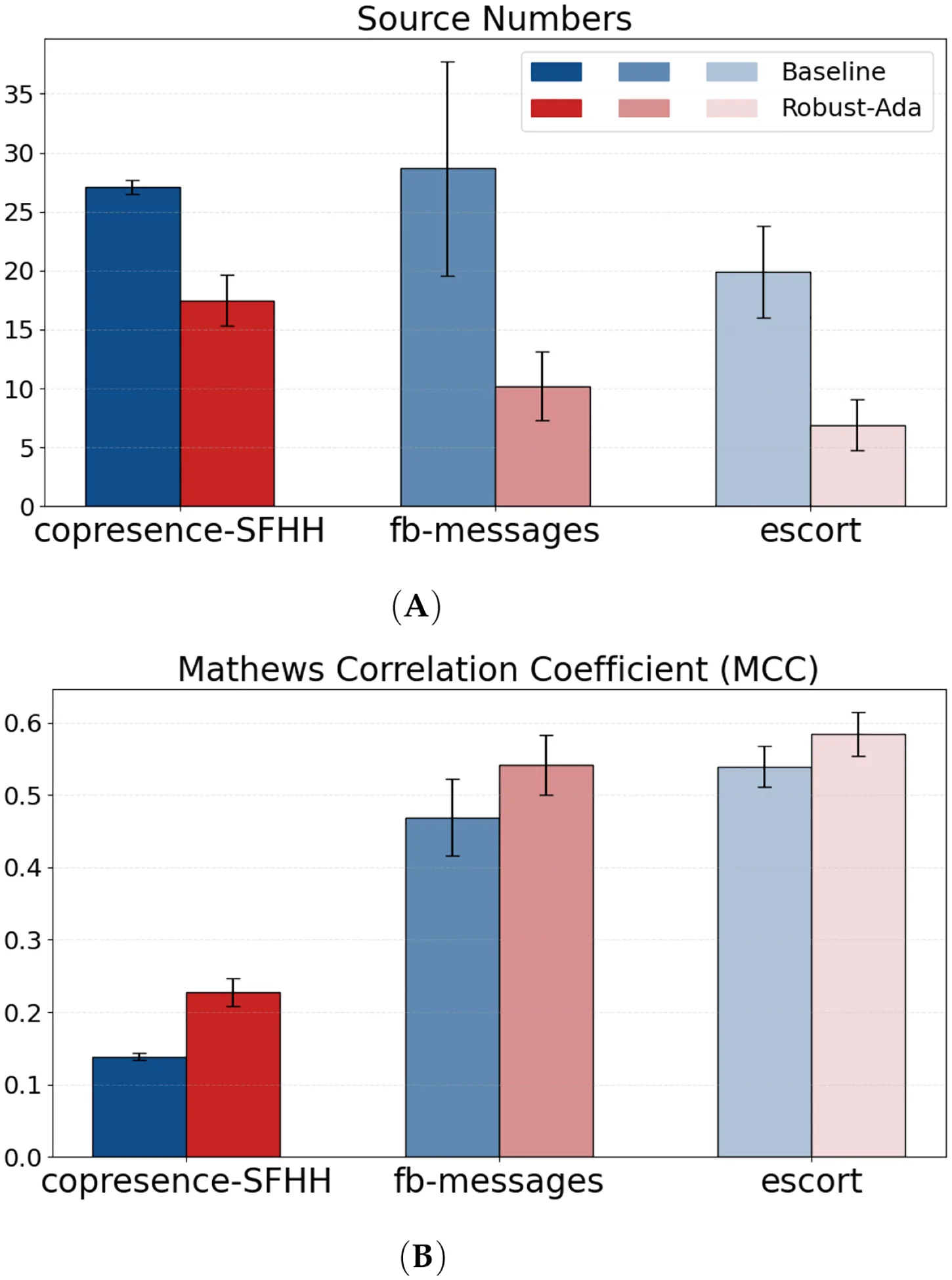
(**A**) Source counts at the end of the online experiment (lower is better: fewer redundant sources and more consistent trees). Each box summarizes 20 runs per dataset (X-axis). *Robust-Ada-Source* (red) yields lower means and smaller dispersion than the baseline (blue), indicating significantly fewer redundant sources. (**B**) Matthews correlation coefficient (MCC; higher is better: fewer false positives/negatives) under the same setting as (**A**). Also, (**B**) share the same legend as (**A**). *Robust-Ada-Source* achieves higher MCC across all datasets, confirming improved accuracy and robustness of the reconstructed trees.

Figure 6A suggests the solid-line source numbers produced by our approach deviate from the dash-line baselines after the second timestamp, and converge to a small number against the unbounded counterparts of the baselines. Note that the ground truth sources of the three datasets are 3, 5, and 10 while each baseline’s source number exaggerates more than at least two times at the seventh timestamp. Figure 7A summarizes the cumulative results as *Robust-Ada-Source* can control the growth of source number on all three datasets under three cross-edge dropout rates. The weight adjustment strategy is more effective given social media data and sexual networks which are relatively sparser over time compared to the contact-network, copresence-SFHH. The baseline method for minimum level-2 approximation of the Steiner tree is neither robust nor optimal given *Robust-Ada-Source*’s outperformance from the same solution space. In contrast, satisfying results of *Robust-Ada-Source* happen under various perturbations, indicating the dynamic cost on source candidates rule out identical solutions with multiple sources as Figure 5. We define the 0% perturbation case as the control group and use it as a control to evaluate our approach for the next.

#### 5.2.2. *Robust-Ada-Source* Prefers Bounded Detours to Control Source Numbers Under Missing-Edges Perturbations While Maintaining Decent MCC Scores Against All Baselines

As seen in Figure 7B, although additional positives by detouring might decrease the MCC, it happens that *Robust-Ada-Source* recovers more false positive cases, which enhances the overall MCC. The MCC score is relatively lower under the dataset of copresence-SFHH which unfortunately suffers from large redundant sources as well. Nevertheless, regardless of the models and data, the model is always better than one without the weight adjustments approach. Our approach tends to detour when feasible paths from the high-risk candidates remain in the 20%-off network compared to increasing the redundant sources often in the 80%-off network. The baselines with perturbation are sensitive to the data and the diffusion model as they struggle to choose a detour or a new source, resulting in inconsistent source numbers with a large deviation.

#### 5.2.3. The Increase in MCC Is Attributed to Robustly Controlled False Positive Cases Aligning Well with Theorems 1 and 2: The Method Is Effective Under Offline Settings

**Figure 8.**
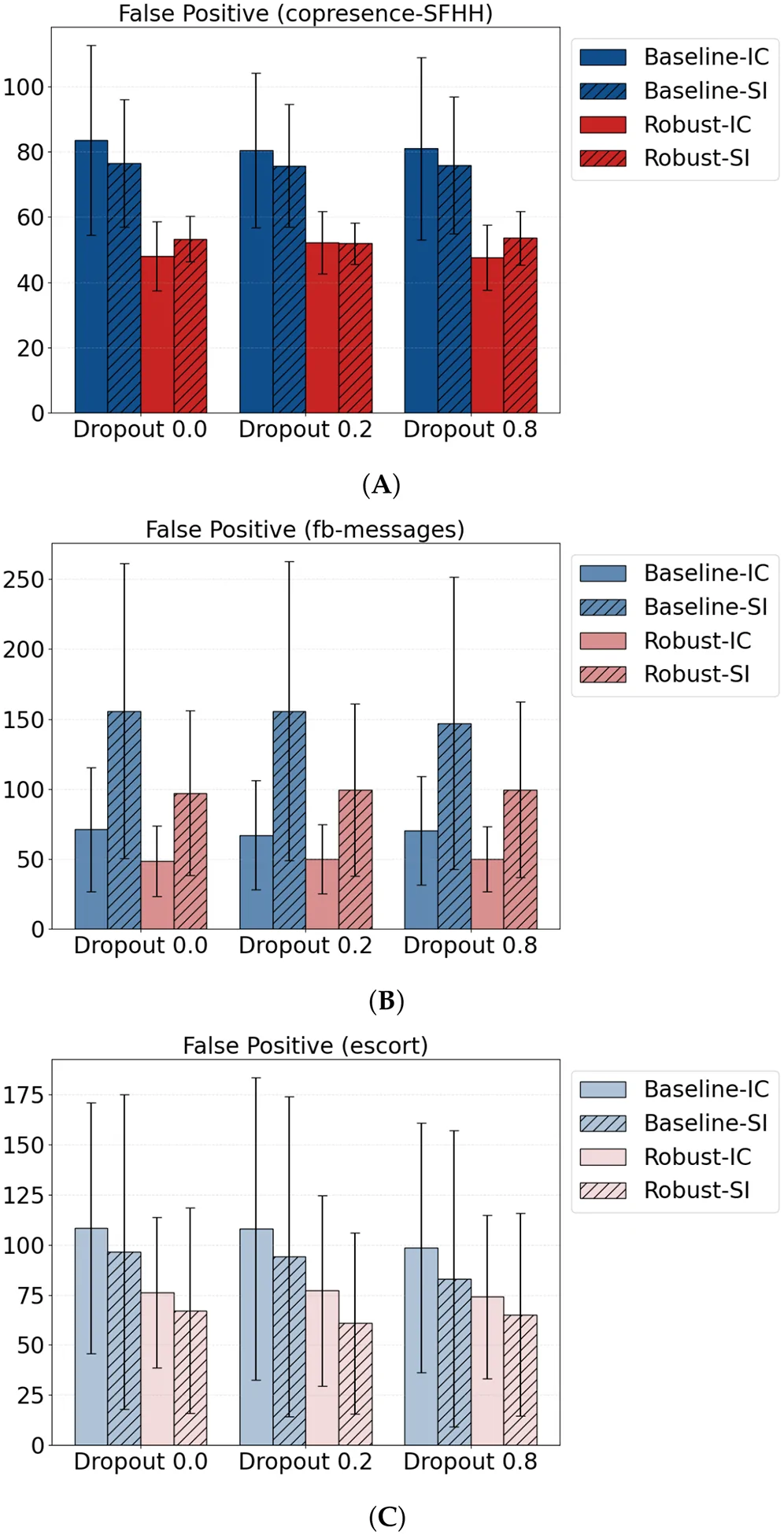
Sensitivity of false positives (FP) to missing-edge rates and diffusion models. We vary edge dropout at 0% (offline, no missing edges), 20% (light), and 80% (heavy), under two simulators (SI and IC), and repeat across three datasets: (**A**) Copresence-SFHH, (**B**) fb-messages, and (**C**) escort. Across all datasets, dropout levels, and models, *Robust-Ada-Source* (red) consistently yields fewer FPs than the baseline (blue). This systematic reduction explains the MCC gains in Figure 7, including in the offline 0% benchmark. The comprehensive tabular results can be founded in Appendix B Table A1.

By Figure 8A–C, the false positive introduced by detouring is always below the counterpart across all datasets and models. The epidemic model with different dropout rates might cause large variations in false positive mistakes but our approach remains insensitive to the models and data selections which further validates the robustness. For example, the boxplots present amplified variance in false positive mistakes among SI models from escort and fb-messages while the variance yielded by *Robust-Ada-Source* has been reduced. Meanwhile, the control case with zero perturbation shows that the method can even improve offline decision-making by reducing the false positive numbers from the reconstruction model. This is due to the elimination of redundant solutions as illustrated in Figure 5.

#### 5.2.4. *Robust-Ada-Source* Yield Lower Variance in Source Numbers, Indicating Better Consistency of Sources

**Figure 9.**
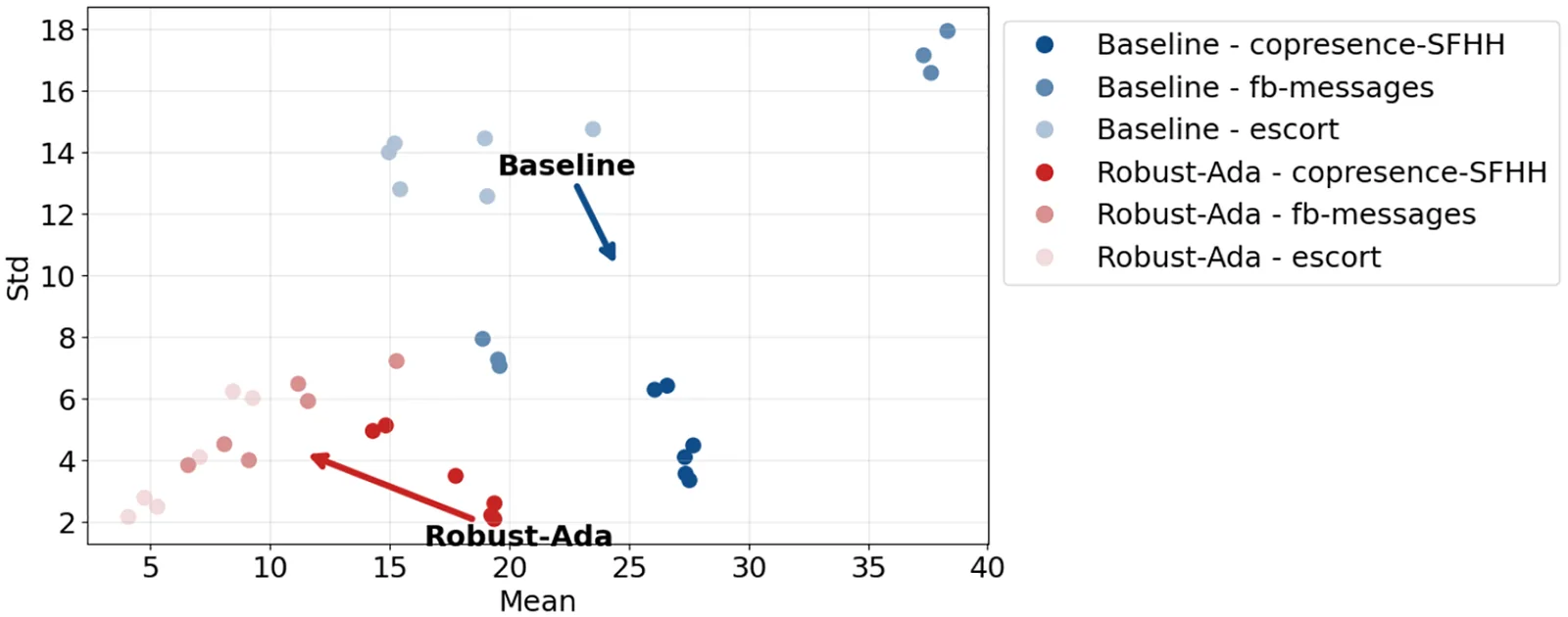
The positive correlation between the means of the source number (X-axis) and the standard deviation (Y-axis). The points at the lower left corner (red) are from *Robust-Ada-Source*.

The overall source variance of *Robust-Ada-Source* is much smaller than the baseline as shown by Figure 9. Especially considering the datasets of fb-messages and escort, the standard deviations forms clusters that are far away between the proposed approach and the baseline. It is interesting to see that the effectiveness of controlling sources remains insensitive to the edge dropout rates. The difference among clusters degenerates when the temporal network has too many isolated components, such as copresence-SFHH, which is the case when all the methods suffer from low MCC and large redundant sources. The above results convey two messages: (1) the adaptivity of *Robust-Ada-Source* depends on the network topology. When the network becomes too sparse, isolating reports, both our method and the baseline are forced to add redundant sources. (2) Each network must have its maximum capacity for dropout to enable bounded detour given random removal of cross-edges. We expected that the performance of our approach could be improved by optimizing the initialization hyperparameter λ and δ given a hypothetical dropout rate through grids-search, which are set constantly as 5000 and 10 respectively, throughout the experiment. The capacity could be further limited if the missing edges are not sampled randomly but adversarially. This could be a future direction to generalize the current formulation.

## 6. Conclusions

We presented a source-robust, non-parametric framework for reconstructing temporal network diffusion under online, incomplete data. The method augments directed-Steiner–based reconstruction with adaptive source costs that widen over time between high- and low-risk candidates, thereby preferring bounded detours over opening redundant sources. Our contributions are threefold: *(i)* a simple, model-agnostic cost-adjustment mechanism that plugs into standard Steiner approximations; *(ii)* theoretical guarantees, including a general upper bound on false-positive regret and a logistic-growth case showing improved constants relative to a no-adjustment baseline; and *(iii)* empirical gains across three real datasets, with roughly twofold fewer redundant sources, ~50% lower source-count variance, and ~5% higher MCC in both offline and online settings.

Nevertheless, the performance of *Robust-Ada-Source* depends on network topology and sparsity: when the time-expanded graph becomes highly fragmented, both our method and baselines are forced to introduce additional sources. The analysis assumes incompleteness concentrated at the most recent timestamp and relies on a level-2 (shortest-path concatenation) Steiner approximation; different approximation regimes may alter constants. Finally, we used fixed hyperparameter (λ,δ) for simplicity; more systematic tuning could further improve accuracy–parsimony trade-offs. In additions, the current code implementation suffers from scalability in a longer window. Ignoring the linear programming (LP) approximation (5), Algorithm 1 runs in $𝒪(T|C||\overset{‾}{K}||\bar{ℰ}|\text{log}|\bar{𝒱}|)$, where bars denote typical per-step sizes. The LP (5) is the bottleneck—standard solvers (e.g., we used Gurobi) become impractical for T≥15; scaling calls for design of sparsity-aware heuristics and data structures.

We see several concrete avenues: *(i)* robustness under adversarial or structured missing-edge processes and delayed reporting; *(ii)* scaling to larger, rapidly evolving social and mobility networks via streaming solvers and graph sparsification; *(iii)* integration with real-time monitoring systems for adaptive hyperparameter selection and alerting; *(iv)* uncertainty quantification over inferred sources and paths (e.g., posterior sampling over Steiner alternatives); and *(v)* extensions to other parametric objectives e.g., deep-learning-based objectives that combines heterogeneous data-driven signals with adaptive costs. Together, these directions would broaden applicability and further stabilize online diffusion reconstruction in noisy, dynamic environments.

## Figures and Tables

**Table 1.** Notations and definitions.

Symbol	Meaning
V	Set of nodes, |V|=N.
E	Set of temporal edges (u,v,t) with u,v∈V and t∈[T]={1,…,T}.
$G_{t}$	Observed temporal network at timestamp t.
${𝒢}_{t}=\left({𝒱}_{t},{ℰ}_{t}\right)$	Time-expanded static graph built from $\left\{G_{1},\ldots ,G_{t}\right\}$.
C⊆V	Candidate source set.
$C_{t}^{H}\subseteq C$	High-risk source candidates at time t (e.g., previously inferred sources).
$R_{t}$	Cumulative report set up to time $t;\psi (t)=\left|R_{t}\right|$.
$K_{t}$	Number of activated nodes at time t used by the Steiner approximation.
${\overset{‾}{P}}_{t}^{c,r},{\overset{‾}{D}}_{t}^{c,r}$	Shortest path and distance in ${𝒢}_{t}$ from candidate c to report r (online graph).
$w_{t}^{\left(z_{c},t_{c}\right),\left(c,t_{c}\right)}$	Dummy-to-candidate edge cost at time t (source-opening cost).
λ>0	Initial penalty. [second round] A high default cost applied when the model adds an extra source, making this choice less favorable than increasing path length (detouring).
δ>0	Lower bound (floor) on source costs. [second round] Acts as a regularizer to prevent over-detouring: the model avoids adding redundant sources or detouring from lower-risk candidates unless the expected cost reduction exceeds δ per action.
$w_{\text{max}}^{t},w_{\text{min}}^{t}$	Maximum/minimum source-edge costs at time t.
α∈(0,1)	Fraction of reports infeasible under missing edges.
$\Psi_{0}(T)=\int_{0}^{T}\psi (s)ds,\Psi_{1}(T)=\int_{1}^{T}\frac{\psi (s)}{s}ds$	Integrals used in regret bounds.
𝒯(T)	Cumulative false-positive regret.

**Table 2.** Descriptive statistics of temporal networks.

Temporal Network	Nodes	Edges	Timestamps	Type
copresence–SFHH	403	2,834,970	55	Contact
fb–messages	1899	123,468	203	Social
escort	13,769	80,880	36	Sexual

Table entries summarize node/edge counts, number of timestamps, and interaction type.

## Data Availability

The source code for reproducing the results and experiments in this study is publicly available at https://github.com/pathology-dynamics/robust_source_ndp (accessed on 10 September 2025). The real-world temporal network datasets used in this study—Copresence-SFHH (accessed on April 2024), fb-messages (accessed on May 2024), and escort networks ((accessed on April 2024))—are available from the data folder and associated references cited within the manuscript.

## Appendix A. Prerequisites and Setups

Let V be a set of nodes with the size as N and t∈[T] be a timestamp from the discrete set [T]={1,2…,T}. Let (u,v,t) be an interaction from u to v at time t,u,v∈V and t∈[T]. We denote the set of all such interactions by E={(u,v,t)∣u,v∈V,t∈[T]}. A network G=(V,E) is an indirect temporal network. We can always transform temporal networks into directed static networks [38]. Given a timestamp t∈[T], we construct a static graph ${𝒢}_{t}=\left({𝒱}_{t},{ℰ}_{t}\right)$ as follows: For every u∈V, we create a series of time-stamp copies $\left(u,\overset{ˆ}{t}\right)$ for every $\overset{ˆ}{t}\leq t$, and denote the whole set ${𝒱}_{t}$. For every $\overset{ˆ}{t}\leq t$, We add an self-edge with zero weight between every $\left(u,\overset{ˆ}{t}\right)$ and $\left(u,\overset{ˆ}{t}+1\right)$ and denote it by $<(u,\overset{ˆ}{t}),(u,\overset{ˆ}{t}+1)>$. For each edge $\left(u,v,\overset{ˆ}{t}\right)$, we add two cross-edges $<\left(u,\overset{ˆ}{t}\right),\left(v,\overset{ˆ}{t}\right)>$ and $<\left(v,\overset{ˆ}{t}\right),\left(u,\overset{ˆ}{t}\right)>$ both with an identical weight 1. We use ${ℰ}_{t}$ to denote the set of all edges created.

A temporal path between any $\left(u,t_{1}\right)$ and $\left(v,t_{2}\right)$ for $t_{1}\leq t_{2}$ is a subset of edges $p\subset E,p=\left\{\left(u,w_{1},t_{1}\right),\left(w_{1},w_{2},t_{2}\right)\ldots \left(w_{n-1},v,t_{n}\right)\right\}$ where $t_{1}\leq t_{2}\ldots \leq t_{n}$. A set of temporal paths $x=\left\{p_{1},p_{2}\ldots \right\}$ spans a subset R⊂V if for each (u,t)∈R there exits counterparts $\left(u,t^{\prime }\right)\in 𝒰(P)$ such that $t^{\prime }\leq t$, where 𝒰 denote the nodes of a set of edges. We begin with the general formulation of [3,5,6] as the closest offline problem. Add dummy nodes $\left(z_{c},t_{c}\right)$ to ${𝒱}_{t}$ each with an edge (the weight to be determined later), $<\left(z_{c},t_{c}\right),\left(c,t_{c}\right)>$, pointing to $\left(c,t_{c}\right)$ added to ${ℰ}_{t}$ for all c∈C, where $t_{c}=\text{arg}\;{\text{min}}_{(c,t)\in V}t$, which is the earliest possible start of a temporal path rooted from c. Let $\lambda \in R^{+}$ be an initialization of source cost as a hyper-parameter. In addition, let $K_{t}=\left|R^{t}\right|$. Consider the following preliminary formulation:

**Definition A1.** *Given a temporal network* ${𝒢}_{t}$, *a set of candidate sources* C⊂V, *a report log $\left.R_{t}=\left\{(u,\overset{ˆ}{t})|u\in {𝒱}_{t},\overset{ˆ}{t}\leq t\right)\right\}$*, *a positive integer* M, *find a set of temporal path* x *such that*

1. ${\text{min}}_{x}\;f\left(x|R_{t},{ℰ}_{t},\lambda \right)$, *where* f *is a distance function*
2. x *spans* $R_{t}$ *and obey the order*.
3. *Sources of* x⊂C.
4. *The number of sources* ≤M.

The above model-independent offline formulation leads to the Minimum Directed Steiner Tree, an NP-hard problem [17]. The best approximation algorithm reaches $𝒪\left(\ell (\ell -1)K_{t}^{1/\ell }\right)$, for recursion parameter ℓ≥2.

*Details of Weights Adjustment*

(A1)
$$w_{t}^{\left(z_{c},t_{c}\right),\left(c,t_{c}\right)}=\text{max}\left(\frac{w_{t-1}^{\left(z_{c},t_{c}\right),\left(c,t_{c}\right)}}{t},\delta \right)c\in C_{t}^{H}$$

All $w_{0}^{\left(z_{c},t_{c}\right),\left(c,t_{c}\right)}=\lambda$ as an initial cost controlling the number of candidates.

## Appendix B. Theorems

### Appendix B.1. Proof of Theorem 1

**Proof.** By definition, the regret is defined as Equation (4)

$$𝒯(T)=\sum_{t=1}^{T}f\left(x|{\overset{‾}{P}}_{t},{\overset{‾}{D}}_{t}\right)-\underset{x_{t}\in 𝒳}{\text{min}}f\left(x_{t}|P_{t},D_{t}\right).$$

Using the structure of the regret under noisy observations, we have:

$$𝒯(T)\leq \sum_{t=1}^{T}\psi (t)\left(\alpha \left(w_{\text{max}}^{t}-1\right)+(1-\alpha )\left(w_{\text{max}}^{t}-w_{\text{min}}^{t}+1\right)\right)\;=\sum_{t=1}^{T}\psi (t)\left(w_{\text{max}}^{t}-\left[2\alpha -1+(1-\alpha )w_{\text{min}}^{t}\right]\right).$$

Assuming $w_{\text{max}}^{t}\leq \frac{\lambda }{t}$ and $w_{\text{min}}^{t}\geq \frac{\delta }{t}$, and approximating the sum by integrals, we discover the regret bound for Robust-Ada-Source as Equation (7):

$$𝒯(T)\leq \int_{1}^{T+1}\psi (s)\cdot \frac{\lambda }{s}ds-[2\alpha -1+(1-\alpha )\delta ]\int_{0}^{T}\psi (s)ds\;=\lambda \;\text{ln}\left(T+1\right)\Psi_{1}\left(T+1\right)-\left[2\alpha -1+\left(1-\alpha \right)\delta \right]\Psi_{0}\left(T\right).$$

For the baseline method, where $w_{\text{max}}^{t}=w_{\text{min}}^{t}=\frac{\lambda }{t}$, we get the counterpart of the basleine as Equation (8):

$$𝒯(T)\leq \sum_{t=1}^{T}\psi (t)\left(\alpha \left(\frac{\lambda }{t}-1\right)+(1-\alpha )\right)\;\leq \int_{1}^{T+1}\psi (s)\cdot \left(\frac{\alpha \lambda }{s}+1-\alpha \right)ds\;=\left[\alpha \lambda \;\text{ln}\left(T+1\right)+1-\alpha \right]\Psi_{1}\left(T+1\right).$$

To compare the two, define the ratio involving α as Equation (6):

$$\frac{𝒯(T{)}^{\text{Robust-Ada-Source}}}{𝒯(T{)}^{\text{baseline}}}\leq \frac{\lambda \;\text{ln}(T+1)\Psi_{1}(T+1)-[2\alpha -1+(1-\alpha )\delta ]\Psi_{0}(T)}{\left[\alpha \lambda \;\text{ln}(T+1)+1-\alpha ]\Psi_{1}(T+1\right)}.$$

Sufficiently, the inequality

$$\frac{\lambda \;\text{ln}(T+1)(1-\alpha )+\alpha -1}{2\alpha -1+(1-\alpha )\delta }\leq \frac{\Psi_{0}(T)}{\Psi_{1}(T+1)}$$

guarantees that *Robust-Ada-Source* performs at least as well.

Now apply L’Hôpital’s Rule to the both sides:

$$\underset{T\to \infty }{\text{lim}}\frac{\Psi_{0}(T)}{\Psi_{1}(T+1)}=\underset{T\to \infty }{\text{lim}}\frac{\Psi_{0}^{\prime }(T)}{\Psi_{1}^{\prime }(T+1)}=1,$$

Then it suffices that:

$$\frac{\lambda (1-\alpha )}{\left(T+1)[2\alpha -1+(1-\alpha )\delta \right]}\leq 1,$$

which is often satisfied for large T, proving the claim. □

### Appendix B.2. Proof of Theorem 2

**Proof.** We let $S_{t}\subseteq R_{t}$ denote the reports’ subject that becomes infeasible under missing edges perturbation at each time t. Each report set $R_{t}$ after perturbation can be divided into two groups: feasible and infeasible. The largest difference among the infeasible group is $w_{\text{max}}^{t}-1$, which is the cost of adding a new source minus the cost of extending from an existing path with one cross-edge. Similarly, the feasible group is with detours from the existing paths, where the gap is bounded by $w_{\text{max}}^{t}-w_{\text{min}}^{t}+1$. This yields the following upper bound:

$$𝒯(T)=\sum_{t=1}^{T}f\left(x|{\overset{‾}{P}}_{t},{\overset{‾}{D}}_{t}\right)-\underset{x^{t}\in 𝒳}{\text{min}}f\left(x_{t}|P_{t},D_{t}\right)\;\leq \;\sum_{t=1}^{T}\left(\underset{\text{infeasible}}{\underbrace{\sum_{S_{t}}\left(w_{\text{max}}^{t}-1\right)}}+\underset{\text{feasible}\;\text{with}\;\text{detours}}{\underbrace{\sum_{R_{t}\setminus S_{t}}\left(w_{\text{max}}^{t}-w_{\text{min}}^{t}+1\right)}}\right)$$

The baseline method with identical weight and no decaying indicating $w_{\text{max}}^{t}=w_{\text{min}}^{t}=\lambda$ which becomes:

$$=\sum_{t=1}^{T}\left(\left|S_{t}\right|\left(w_{\text{max}}^{t}-1\right)+\left|S_{t}\right|-\left|R_{t}\right|\right)$$

If taking infinite sum assuming T→∞, we discover the bound for the baseline as Equation (9);

$$\leq (\lambda -1)\int_{1}^{T+1}\psi (s)ds\;\leq (\lambda -1)𝒪\left(\int_{1}^{T+1}\frac{1}{1+\text{exp}(-\tau (t-T/2))}ds\right)\;\leq (\lambda -1)𝒪(T)$$

In contrast, our method will control the gap among w by the regularization parameter, $w_{\text{min}}^{t}\geq \delta$. We assume a linear perturbation rate for infeasible reports: $\left|S_{t}\right|=\alpha \psi (t)$. We can expand the above inequality and discovered the bound for Robust-Ada-Source (10):

$$\sum_{t=1}^{T}\left(\underset{\text{infeasible}}{\underbrace{\sum_{S_{t}}\left(w_{\text{max}}^{t}-1\right)}}+\underset{\text{feasible}\;\text{with}\;\text{detours}}{\underbrace{\sum_{R_{t}\setminus S_{t}}\left(w_{\text{max}}^{t}-w_{\text{min}}^{t}+1\right)}}\right)\;=\sum_{t=1}^{T}\left(\left|S_{t}\right|\left(\left(w_{\text{max}}^{t}-1\right)+\left|R_{t}\setminus S_{t}\right|\left(w_{\text{max}}^{t}-w_{\text{min}}^{t}+1\right)\right)\right.\;=\sum_{t=1}^{T}\left|R_{t}\right|\left(\alpha \left(w_{\text{max}}^{t}-1\right)+(1-\alpha )\left(w_{\text{max}}^{t}-w_{\text{min}}^{t}+1\right)\right)\;=\sum_{t=1}^{T}\left|R_{t}\right|\left(w_{\text{max}}^{t}-2\alpha +1-(1-\alpha )w_{\text{min}}^{t}\right)\;\leq \left(\lambda -2\alpha +1-(1-\alpha )\delta \right)\int_{1}^{T+1}\psi (s)ds\;\leq (\lambda -2\alpha +1-(1-\alpha )\delta )𝒪(T)$$

To make sure:

λ−1≥λ−2α+1−(1−α)δ

We must have:

$$2=\frac{2\alpha -2}{\alpha -1}\leq \delta$$

□

### Appendix B.3. Level-2 Is a $O\left(\sqrt{{\text{K}}_{\text{t}}}\right)$ *Approximation*

**Theorem A1.** *The Formulation* (5) *reaches* $𝒪\left(\sqrt{K_{t}}\right)$ *approximation*.

The above Theorem A1 confirms that solving the problem will yield a level-ℓ=2 approximation. However, the formulation’s source number and false positive cases remain sensitive to online data. Even without edges dropout, the level-2 approximation cannot distinguish single and multiple-source Steiner trees when their total distance equals each other, see Figure 5.

**Proof.** The formulation of concatenating the shortest paths for level-2 approximations of the minimum directed Steiner tree is identical to the formulation of Rozenshtein et al. [5] from Proposition 7, which yields $𝒪\left(\sqrt{K_{t}}\right)$ approximation. □

### Appendix B.4. Level-2 Approximation Can Be Solved by Linear Programming

**Theorem A2** (Time-interval condition for the time-expanded network). *Let* G=(V,E) *be a temporal network with interactions* (u,v,t)∈E *for* t∈[T]={1,…,T}. *Fix* t∈[T] *and construct the time-expanded directed graph* ${𝒢}_{t}=\left({𝒱}_{t},{ℰ}_{t}\right)$ *as follows*:

1. *For each* u∈V *and each* $\overset{ˆ}{t}\in \{1,\ldots ,t\}$, *create a node* $(u,\overset{ˆ}{t})\in {𝒱}_{t}$.
2. *For each* u∈V *and* $\overset{ˆ}{t}\in \{1,\ldots ,t-1\}$, *add the forward “stay” arc* $\left\langle (u,\overset{ˆ}{t}),(u,\overset{ˆ}{t}+1)\right\rangle$ *to* ${ℰ}_{t}$.
3. *For each $(u,v,\overset{ˆ}{t})\in E$ with* $\overset{ˆ}{t}\leq t$, *add the same-time cross arcs* $\left\langle (u,\overset{ˆ}{t}),(v,\overset{ˆ}{t})\right\rangle$ *and* $\left\langle (v,\overset{ˆ}{t}),(u,\overset{ˆ}{t})\right\rangle$ *to* ${ℰ}_{t}$.

*Let* $𝒰\left(R_{t}\right)$ *be the set of reported events up to time* t, *and associate each report* $r\in 𝒰\left(R_{t}\right)$ *with a unique vertex* $\left(u_{r},\tau (r)\right)\in {𝒱}_{t}$ *at its timestamp* τ(r)∈{1,…,t}.

*Then every directed path* P *in* ${𝒢}_{t}$ *visits report timestamps in a nondecreasing order, and moreover the set of reports encountered by* P *has consecutive timestamps: if* P *visits two reports* $r_{a},r_{b}$ *with* $\tau \left(r_{a}\right)\leq \tau \left(r_{b}\right)$, *then for every* $\overset{ˆ}{t}$ *with* $\tau \left(r_{a}\right)\leq \overset{ˆ}{t}\leq \tau \left(r_{b}\right),P$ *visits some vertex at time* $\overset{ˆ}{t}$, *and hence any report located at a time strictly between* $\tau \left(r_{a}\right)$ *and* $\tau \left(r_{b}\right)$ *that lies on* P *is also visited by* P. *Consequently, when rows of the incidence matrix are ordered by nondecreasing* τ(⋅), *each column corresponding to a path has its* 1-*entries in a single contiguous block*; *i.e*., *the matrix has the consecutive-ones property (by columns)*.

**Proof.** By construction, the only arcs that change the time index are the forward “stay” arcs $\left\langle (u,\overset{ˆ}{t}),(u,\overset{ˆ}{t}+1)\right\rangle$, which strictly increase time by +1. All other arcs are same-time cross arcs that keep the time index unchanged. Hence along any directed path P in ${𝒢}_{t}$, the time component of visited vertices is a *nondecreasing* sequence. In particular, P cannot move backward in time.

Suppose P visits two reports $r_{a}=\left(u_{r_{a}},\tau \left(r_{a}\right)\right)$ and $r_{b}=\left(u_{r_{b}},\tau \left(r_{b}\right)\right)$ with $\tau \left(r_{a}\right)\leq \tau \left(r_{b}\right)$. Between these two visits, the path can only use same-time cross arcs (which do not change the time index) and forward “stay” arcs (which increase the time index by exactly 1). Therefore, for every integer $\overset{ˆ}{t}$ with $\tau \left(r_{a}\right)\leq \overset{ˆ}{t}\leq \tau \left(r_{b}\right)$, the path P contains at least one vertex whose time component equals $\overset{ˆ}{t}$. Equivalently, the multiset of timestamps of vertices visited by P contains the full interval $\left\{\tau \left(r_{a}\right),\tau \left(r_{a}\right)+1,\ldots ,\tau \left(r_{b}\right)\right\}$.

Now consider the incidence row vector for any report $r=\left(u_{r},\tau (r)\right)$. The column of the constraint matrix corresponding to path P has entry 1 in row r if and only if P passes through the report vertex $\left(u_{r},\tau (r)\right)$. From the argument above, whenever P visits reports at times $\tau \left(r_{a}\right)$ and $\tau \left(r_{b}\right)$ with $\tau \left(r_{a}\right)\leq \tau \left(r_{b}\right)$, the set of report *timestamps* encountered by P is a contiguous interval in $\left\{\tau \left(r_{a}\right),\ldots ,\tau \left(r_{b}\right)\right\}$. Consequently, if we order all report-rows by nondecreasing timestamp τ(⋅), then the 1-entries in the column for P occur on a single contiguous block of rows. This is exactly the consecutive-ones property (by columns).

Thus every column of the report-incidence matrix has consecutive ones after sorting rows by time, proving the claim. □

**Corollary A1** (TU and integrality via C1P). *Let A be the report-incidence matrix whose $\left(r,\left(c,r^{\prime }\right)\right)$*-*entry equals* $1\left(r\in {\overset{‾}{P}}^{c,r^{\prime }}\right)$, *with rows ordered by nondecreasing* τ(r). *By Theorem A2, A has the consecutive-ones property (by columns); hence A is totally unimodular. Adding bound constraints* 0≤x≤1 *appends* ±I *blocks, and converting* Ax≥1 *to* (−A)x≤−1 *preserves total unimodularity. Therefore the full constraint matrix of Problem* (5) *is TU, and because the right-hand side is integral, the LP relaxation has an integral polyhedron; in particular, the LP and IP share the same optimal solutions*.

### Appendix B.5. An Adversarial Scenario Will Always Cause a Linear Error Lower Bound

**Theorem A3.** *If at least one report becomes infeasible at every* t, *solving the minimization problem defined in* Equation (5) *will yield a lower bound on the regret (on false positives) as*:

(A2)
𝒯(T)=Ω(T)

**Proof.** For the lower bound, we assume an adversarial scenario. By the minimax inequality, we have:

$$𝒯(T)=\sum_{t=1}^{T}f\left(x|{\overset{‾}{P}}_{t},{\overset{‾}{D}}_{t}\right)-\underset{x^{t}\in 𝒳}{\text{min}}\;\underset{P_{t},D_{t}}{\text{max}}f\left(x_{t}|P_{t},D_{t}\right)\;\geq \sum_{t=1}^{T}f\left(x|{\overset{‾}{P}}_{t},{\overset{‾}{D}}_{t}\right)-\underset{P_{t},D_{t}}{\text{max}}\;\underset{x^{t}\in 𝒳}{\text{min}}f\left(x_{t}|P_{t},D_{t}\right)\;=\sum_{t=1}^{T}\underset{{\overset{‾}{P}}_{t},{\overset{‾}{D}}_{t}}{\text{min}}\left(f\left(x|{\overset{‾}{P}}_{t},{\overset{‾}{D}}_{t}\right)-\underset{x^{t}\in 𝒳}{\text{min}}f\left(x_{t}|P_{t},D_{t}\right)\right)$$

We further assume that the perturbation should yield at least one infeasible report:

$$\geq \sum_{t=1}^{T}w_{\text{max}}^{t}-\left(w_{\text{max}}^{t}-1\right)\;=\sum_{t=1}^{T}1\;=T$$

□

## Appendix C. Additional Experiment Results

We present the complementary summary tables and figures for all the experiments in Section 5.

**Table A1.** Mean ± standard deviation of MCC and number of sources at the final timestamp. Each entry aggregates 20 runs per setting. Dropout rates indicate the fraction of missing edges at the latest timestamp; 0% is the offline benchmark.

Dataset	Model	Approach	Dropout	MCC	Sources
Copresence–SFHH	IC	Robust–Ada	0%	0.257 ± 0.090	14.30 ± 4.96
			20%	0.221 ± 0.071	17.76 ± 3.50
			80%	0.253 ± 0.090	14.84 ± 5.14
			0%	0.146 ± 0.133	26.60 ± 6.43
		Baseline	20%	0.133 ± 0.101	27.69 ± 4.49
			80%	0.145 ± 0.127	26.08 ± 6.30
			0%	0.211 ± 0.039	19.25 ± 2.22
	SI	Robust–Ada	20%	0.214 ± 0.040	19.38 ± 2.61
			80%	0.209 ± 0.045	19.37 ± 2.10
			0%	0.135 ± 0.072	27.37 ± 3.57
		Baseline	20%	0.134 ± 0.069	27.53 ± 3.36
			80%	0.138 ± 0.082	27.34 ± 4.11
fb–messages	IC	Robust–Ada	0%	0.585 ± 0.064	6.58 ± 3.85
			20%	0.593 ± 0.076	8.09 ± 4.53
			80%	0.495 ± 0.080	9.12 ± 4.01
		Baseline	0%	0.517 ± 0.098	18.89 ± 7.95
			20%	0.540 ± 0.104	19.53 ± 7.28
			80%	0.533 ± 0.095	19.60 ± 7.07
	SI	Robust–Ada	0%	0.503 ± 0.102	11.18 ± 6.49
			20%	0.504 ± 0.102	11.59 ± 5.93
			80%	0.493 ± 0.097	15.29 ± 7.23
		Baseline	0%	0.412 ± 0.141	37.32 ± 17.16
			20%	0.416 ± 0.146	37.63 ± 16.59
			80%	0.420 ± 0.134	38.32 ± 17.95
escort	IC	Robust–Ada	0%	0.629 ± 0.058	9.28 ± 6.03
			20%	0.629 ± 0.079	7.06 ± 4.11
			80%	0.627 ± 0.100	8.45 ± 6.25
		Baseline	0%	0.576 ± 0.082	23.50 ± 14.76
			20%	0.593 ± 0.100	19.08 ± 12.58
			80%	0.608 ± 0.116	18.98 ± 14.46
	SI	Robust–Ada	0%	0.572 ± 0.091	4.08 ± 2.17
			20%	0.567 ± 0.091	4.75 ± 2.79
			80%	0.552 ± 0.086	5.29 ± 2.50
		Baseline	0%	0.522 ± 0.113	14.97 ± 14.01
			20%	0.515 ± 0.109	15.21 ± 14.30
			80%	0.529 ± 0.107	15.44 ± 12.81

Entries are reported as mean ± standard deviation.
